# Clinical Validation of the Belay Ascent™ Test to Report on Chromosomal Arm-Level Aneuploidy and Gene-Level Copy Number Variants in Cerebrospinal Fluid Using Low-Pass Whole-Genome Sequencing

**DOI:** 10.3390/cancers18081277

**Published:** 2026-04-17

**Authors:** Qian Nie, Kala F. Schilter, Alexandra Larson, Vindhya Udhane, Viriya Keo, Sakshi Khurana, Jennifer N. Adams, Anthony Acevedo, Daniel Sanchez, Tarin Peltier, Kathleen Mitchell, DeElegant Robinson, Kyle M. Hernandez, Christopher Douville, Chetan Bettegowda, Honey V. Reddi

**Affiliations:** 1 Belay Diagnostics, 1375 W. Fulton St., Chicago, IL 60607, USA; snie@belaydiagnostics.com (Q.N.); kschilter@belaydiagnostics.com (K.F.S.);; 2 Johns Hopkins University School of Medicine, Baltimore, MD 21205, USA; cdouvil1@jhmi.edu (C.D.); cbetteg1@jhmi.edu (C.B.)

**Keywords:** low-pass whole genome sequencing, gene amplifications and deletions, CSF liquid biopsy

## Abstract

Background: Evaluation of chromosome aneuploidy and gene-level copy number alterations for diagnosis, prognosis, and therapeutic decision-making in solid tumors is the standard of care. Chromosomal microarray (CMA), next-generation sequencing (NGS), immunohistochemistry (IHC), and fluorescence in situ hybridization (FISH) are the gold standard for detecting these variants in tumor tissue. In contrast to most solid tumors, cancers of the central nervous system (CNS) pose a unique challenge for effective detection via plasma due to the blood–brain barrier (BBB), with the additional challenges of brain biopsy or surgery being highly invasive and posing a significant risk to the patient. The Belay Ascent™ liquid biopsy test uses low-pass whole-genome sequencing (LP-WGS) to report on chromosome arm-level aneuploidy and gene-level copy number variants (CNVs) in cerebrospinal fluid (CSF) to inform diagnosis, prognosis, and therapeutic decision-making in CNS tumors. Methods: This study presents the equivalence of Ascent™ in detecting chromosome arm-level aneuploidy and gene-level CNVs using 48 tissue specimens followed by a clinical validation using a cohort of 32 CSF specimens with matched tissue-based tumor profiling information. Results: Equivalence of Ascent™ in detecting chromosome arm-level aneuploidy and gene-level CNVs using 48 tissue specimens was shown to have 100% and 97% positive percent agreement (PPA), respectively, compared to the gold standard of CMA/NGS. The validation cohort of 32 CSF specimens demonstrated 78% and 90% PPA for aneuploidy and gene-level CNVs, respectively. Clinical impact of Ascent™ was demonstrated, with 243 production cases able to inform the diagnosis and management of CNS tumors with high accuracy. Conclusions: Given the paucity of cells in CSF, limiting the use of karyotyping, CMA, IHC, and FISH, the Belay Ascent™ test provides a highly sensitive novel minimally invasive method for the evaluation of chromosome aneuploidy and gene-level CNVs in CSF.

## Simple Summary

Chromosomal and gene alterations are crucial for cancer diagnosis, classification, and treatment selection, which are potentially identified through tumor tissue testing when feasible. For brain and spinal cord cancers, surgery poses significant patient risks, and plasma-based liquid biopsy tests often fail due to the blood–brain barrier, which restricts tumor-derived DNA from entering the bloodstream. This study evaluates the Belay Ascent™ liquid biopsy test, which analyzes tumor-derived DNA in cerebrospinal fluid. This test detects chromosome arm-level changes and gene-level alterations through low-pass whole-genome sequencing. The results indicate that the test effectively identifies genomic changes, demonstrating that Belay Ascent™ provides a minimally invasive alternative to biopsy or surgery and helps inform diagnosis, prognosis, and therapeutic decision-making in primary and metastatic central nervous system cancers.

## 1. Introduction

Aneuploidy is an abnormal chromosome number resulting from the gain or loss of whole chromosomes or large chromosomal segments relative to the normal euploid state. It is highly prevalent in human disease, serving as the leading chromosomal abnormality in spontaneous miscarriages, congenital disorders such as trisomy 21, and the vast majority of solid tumors [1,2]. Over 88–90% of solid cancers display whole-chromosome or large-segment aneuploidy, making it a near-universal feature of malignancy [3,4]. In the central nervous system (CNS), chromosomal aneuploidy plays a critical role in the pathogenesis, classification, and prognosis of several neoplastic and non-neoplastic neurological diseases. Diffuse gliomas, embryonal tumors, and other primary CNS neoplasms are now defined or subtyped by copy-number changes such as 1p/19q codeletion, chromosome 7 gain/10 loss, and 17p deletion/17q gain, which correlate with prognosis and treatment response and are incorporated into the World Health Organization (WHO) 2021 CNS tumor classification, integrating molecular and histologic features [5]. In tumors with high leptomeningeal dissemination risk, including medulloblastoma, primitive neuroectodermal tumors (PNETs), and ependymoma, additional aneuploid patterns (e.g., 1q gain, 22q loss) predict dissemination and outcome [6]. In contrast, leptomeningeal metastases originating from systemic tumors, such as breast [7], lung [8], and melanoma, retain core driver alterations but develop site-type-specific aneuploidy profiles that inform prognosis and guide targeted therapy. Across both primary and metastatic CNS diseases, aneuploidy reflects underlying chromosomal instability, which drives tumor heterogeneity [9,10] and disease progression and prognosis [7,8]. More broadly, National Comprehensive Cancer Network (NCCN) guidelines recommend molecular profiling of solid tumors to identify actionable genomic alterations, including copy-number changes associated with aneuploidy, for prognostic evaluation and clinical trial enrollment [11,12,13]. Under NCCN CNS guidelines, recurrent arm-level and whole-chromosome alterations such as chromosome 7 gain/10 loss, whole-arm 1p/19q codeletion, and broad copy number imbalance patterns are incorporated into tumor classification, WHO grading, and risk stratification. Although not directly actionable, these aneuploidy signatures guide expected therapeutic response, disease aggressiveness, and eligibility for biomarker-driven clinical trials [14,15].

In addition to chromosome loss/gain, gene-level copy number variants (CNVs) of oncogenic driver genes, particularly amplifications, are considered actionable, as drugs are being developed to target them either via the amplified protein using antibodies or by directly targeting the amplified DNA itself with triplex-forming oligonucleotides (TFOs) or gene-editing tools such as CRISPR/CAS9 [16]. A consequence of gene amplification is the accelerated progression of malignancy, developing a resistance to drugs [17]. Targeted therapies for specific genomic amplifications, such as trastuzumab for human epidermal growth factor receptor 2 (HER2 aka *ERBB2*) and tyrosine kinase inhibitors (TKIs) for *EGFR* amplification, are the most well-known [16]. To determine actionability for cancer, detection of focal gene amplifications in tumor tissue using immunohistochemistry (IHC) and/or fluorescence in situ hybridization (FISH) has become the standard of care [18]; however, these methodologies are less effective or not amenable to being performed in cerebrospinal fluid (CSF) [19]. Loss and deletion of tumor suppressor genes such as *TP53*, *PTEN*, *RB1*, and *CDKN2A* and *CDKN2B* are also clinically relevant, as they drive tumor progression and influence prognosis and therapeutic response [20,21,22]. While deletions are less frequently directly targetable, their identification is recommended in the NCCN guidelines for molecular profiling, where they inform treatment resistance, prognostic assessment, and eligibility for specific therapeutic strategies [15,23].

Accurate detection of chromosome aneuploidy and focal gene CNVs in CNS cancers is therefore essential for diagnosis, prognosis, risk stratification, and therapeutic decision-making in clinical care. CSF represents a minimally invasive and clinically accessible specimen for the molecular evaluation of CNS pathology [24]. Several methodologies (Table 1) like karyotyping, FISH, IHC [19], NGS-based methods, and CMA are employed for clinical aneuploidy detection in prenatal diagnostics and oncology, each with distinct advantages and limitations [25,26,27]. Karyotyping provides full visualization of chromosomes and can detect large structural changes and balanced rearrangements; however, it is cell-culture-dependent and is generally impractical for the low-cellularity samples typical of CSF [28]. FISH offers rapid, culture-free detection of specific aneuploidies, such as 1p/19q co-deletions in gliomas, but its genome coverage is limited to predefined loci and requires viable cells [29,30], similar to IHC, which primarily detects protein expression rather than direct genomic gene-level CNVs [19]. CMA and NGS-based low-pass whole-genome sequencing (LP-WGS) represent advanced tools for aneuploidy and CNV detection in liquid biopsy specimens [7,28,31]. While CMA delivers high-resolution (>50 kb), genome-wide CNV profiling from low-tumor-content tissue, it lacks sensitivity in CSF cell-free DNA (cfDNA) due to fragmentation challenges [32,33].

The use of NGS to evaluate chromosome arm-level loss/gain (aneuploidy) and gene-level CNVs in solid tumors [34] has naturally progressed to evaluating cfDNA in peripheral blood (plasma) [35] to aid in minimally invasive and quicker therapeutic decision-making. One approach uses sequence-specific primers to amplify regions throughout the genome. By evaluating the read depth at each locus, the chromosome arm gains and losses and the degree of aneuploidy can be calculated. Several examples include the Fast Aneuploidy Screening Test Sequencing System (FAST-SeqS) [36,37], which amplifies long-interspersed nucleotide elements (LINEs); the Repetitive Element Aneuploidy Sequencing System (RealSeqS) [38], which amplifies Alu elements; and others that amplify bespoke panels [39,40]. Amplicon-based approaches like these offer many advantages, including a simplified workflow that does not require library construction, higher coverage at the loci queried, and reduced DNA input requirements. LP-WGS resolves this issue by performing unbiased sampling across the entire genome with resolution down to a few kb, which is needed for CSF tumor-derived DNA (tDNA) cancer diagnostics [31,41,42].

To address challenges associated with the sensitivity of plasma due to the blood–brain barrier (BBB) [43,44], Belay developed a portfolio of CSF liquid biopsy tests to inform diagnosis and management of primary and metastatic CNS cancers [45,46,47]. Summit™ 2.0 is a comprehensive genomic profiling (CGP) test that evaluates single-nucleotide variants, gene-level copy number variants, fusions, tumor mutation burden (TMB), and microsatellite instability (MSI) [45]. Ascent™ [46] and Vantage™ [47] leverage the previously described MethySaferSeqS duplex sequencing methodology [48] to enable robust detection of genomic (chromosome arm-level copy number (CN) variants (loss and/or gain) [46] and focal gene alterations) and epigenomic (*MGMT* promoter methylation status) features, respectively, using LP-WGS. The analytical and clinical validation of Ascent™ in detecting chromosome arm-level loss and gain in CSF has been previously published [46]. This study presents additional data on the clinical validation of Ascent™ by demonstrating the equivalence and concordance of this methodology in detecting chromosome arm loss/gain and gene-level CNVs in CSF compared to the gold standard of CMA/NGS/IHC/FISH using CNS tumor tissue and tumor-informed CSF specimens, respectively.

## 2. Materials and Methods

### 2.1. Specimen Cohort

**Figure 1:**
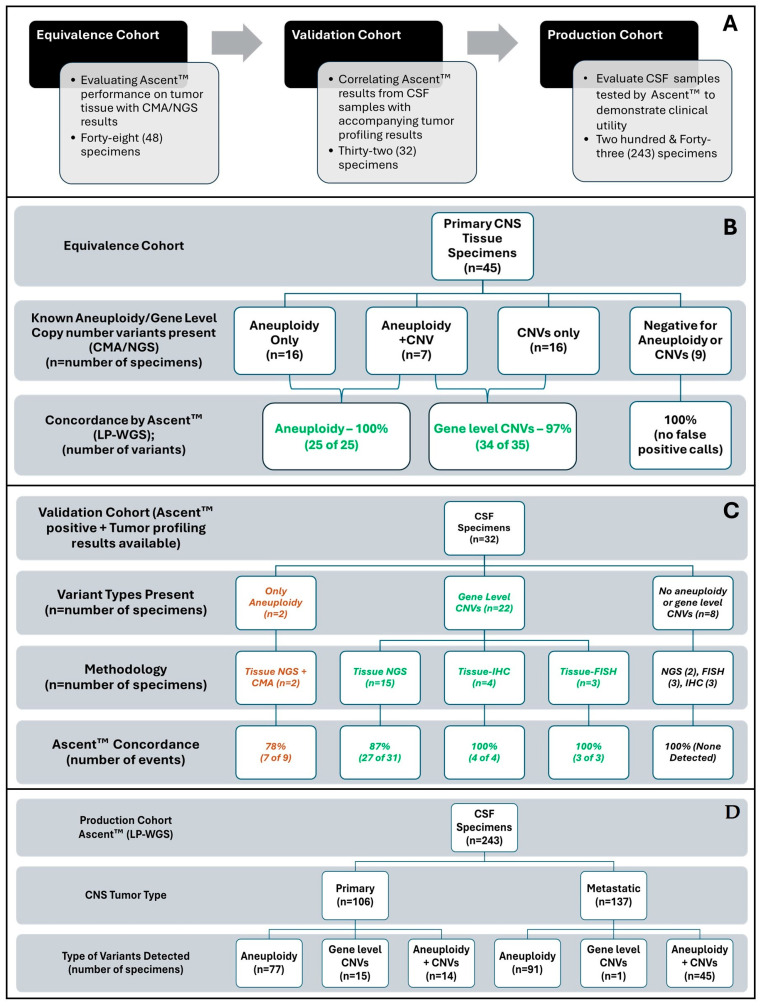
Schema of study (**A**). Equivalence (**B**), validation (**C**), and production cohort specifics (**D**) with concordance results.

A total of 323 unique samples were used in this study (Figure 1A). The equivalence cohort included 48 tissue specimens, including 45 formalin-fixed paraffin-embedded (FFPE) samples from primary CNS tumors and three (3) products of conception tissues with known chromosome arm-level aneuploidy and/or gene-level copy number variants. Twenty-three specimens were obtained under a material transfer agreement with Northwestern University under an IRB (STU00095863) in compliance with the principles of the Declaration of Helsinki and the Health Insurance Portability and Accountability Act (HIPAA), and the remaining 24 were commercially obtained de-identified samples with no associated clinical information (LabCorp, Burlington, NC, USA). The validation cohort included 32 CSF specimens with known tumor profiling results (aneuploidy and gene-level CNVs), which were obtained using samples received from multiple institutions across the US for testing with Summit™, that were de-identified prior to inclusion in this study. The production cohort evaluated 243 Ascent™-positive specimens of the 867 received for clinical testing using both Summit™ [45,46] and Ascent™. Figure 1 also presents the number of specimens/variant types detected in each analysis cohort. This study was conducted under an IRB (Advarra, Pro00078800) in compliance with the principles of the Declaration of Helsinki and HIPAA.

### 2.2. Analysis of Sequencing Data from Ascent™

Analytical validation of Ascent™ for calling chromosome arm-level aneuploidy at an abs(log2r) threshold of 0.09 demonstrated a sensitivity/PPA of 91% and a specificity/NPA of 99%, with a limit of input of ≥20 ng of CSF tDNA [46]. Post-sequencing, data from specimens that passed all established QC metrics were evaluated for chromosome arm-level aneuploidy and gene-level CNVs after demultiplexing and alignment per established threshold cut-offs [46]. The cut-off for aneuploidy calls was abs(log2r) > 0.09 based on the number of copies relative to the panel of normals [46]. For gene-level CNVs, a seg. mean (average number of copies of a specific DNA segment) cut-off value of ≥0.1 was set for gain (amplifications), and ≤0 to <0.1 was set for no CN change, with cut-off values for gene-level deletions being set at ≥−0.2 for loss (deletions) using clinically validated gene-specific quantitative polymerase chain reaction (qPCR) tests.

The results were presented in terms of positive percent agreement (PPA) and negative percent agreement (NPA) to establish concordance for equivalence and clinical validation. PPA in the equivalence and validation cohorts was calculated as a percentage based on the number of aneuploidy or CNV events detected by Ascent™ compared to the number of events known to be present based on tumor profiling information, establishing the true positive rate or sensitivity. NPA in the equivalence and validation cohorts was calculated as a percentage based on the number of aneuploidy or CNV events detected by Ascent™ compared to the number of events known to be present based on tumor profiling information, establishing the false positive rate or specificity. Overall, the PPA and NPA of Ascent™ were calculated by combining results from both cohorts across 80 specimens.

### 2.3. Equivalence of Ascent™ to CMA/NGS for the Detection of Chromosome Arm-Level Aneuploidy and Gene-Level CNVs in Tissue

Forty-eight tissue samples with known chromosome arm-level aneuploidy and/or gene-level CNVs as evaluated by CMA and NGS, respectively, were processed through Ascent™. The accuracy of Ascent™ in calling chromosomal arm-level aneuploidy (loss/gain) and focal gene-level CNVs (amplification/deletion) in tissue was evaluated to establish equivalence compared to CMA and NGS.

### 2.4. Validation of Ascent™ to Detect Chromosome Arm-Level Aneuploidy and Gene-Level CNVs in CSF

Of the 867 specimens received for clinical testing of Summit™ and Ascent™, 345 specimens were negative for both tests, 233 were negative for Ascent™ and positive for Summit™, and 16 specimens failed testing and were not included in this analysis. Of 275 specimens that were positive for Ascent™, 32 CSF specimens included accompanying tissue-based tumor profiling results from CMA/NGS/FISH/IHC, and data from these specimens were evaluated in the validation cohort. The accuracy of Ascent™ in calling chromosome arm-level and gene-level CNVs in CSF was determined by comparing Ascent™ calls to accompanying tumor profiling results. Specimens included 4 primary and 28 metastatic (16 breast, 10 lung, 1 esophageal, and 1 sarcoma) CNS cancers. Gene-level CNVs for *ERBB2* amplification, *EGFR* amplification, and *CDKN2A/2B* deletion detected by Ascent™ were additionally confirmed using a clinically validated gene-specific qPCR assay for each gene.

### 2.5. Demonstrating Clinical Impact of Ascent™ to Detect Chromosome Arm-Level Aneuploidy and Gene-Level CNVs in CSF

To determine the impact of Ascent™ in informing the diagnosis and management of CNS cancers, post-validation, 243 (of 275) specimens with positive results for Ascent™, including both primary and metastatic CNS cancers, chromosome arm-level aneuploidy, and gene-level CNVs detected, were evaluated for clinical significance in terms of WHO diagnosis, actionability (inform medical decision-making), and prognostic impact in the context of tumor type.

## 3. Results

### 3.1. Ascent™ Performance Is Equivalent to CMA/NGS in the Detection of Chromosome Arm-Level Aneuploidy and Gene-Level CNVs in Tissue

Evaluation of 45 primary CNS FFPE tissue specimens with known chromosome aneuploidy (arm-level loss/gain) and gene-level CNVs was processed using Ascent™; all known aneuploidy events were detected (25 of 25) for chromosome arm-level aneuploidy, and 34 of 35 gene-level CNVs were detected, demonstrating 100% and 97% PPA, respectively, for each variant type (Table 2, Figure 1B). In the additional three products of conception tissue specimens, ASEV14, ASEV15, and ASEV48 with complex genotypes (high chromosomal instability), Ascent™ detected a total of 84 chromosome arm-level aneuploidy events (Table 2). The single CNV variant missed by Ascent™ in the FFPE tissue (ASEV-039), a loss of *CDKN2A* and *CDKN2B,* was evaluated by qPCR and confirmed not to be deleted. The discrepancy in results could not be clarified at the source, resulting in one false negative call. The accurate calling of this variant in 16 other cases in the cohort confirmed the ability of Ascent™ to detect gene-level deletions, particularly *CDKN2A* and *CDKN2B* loss, with high sensitivity/accuracy in tissue. No aneuploidy or gene-level CNV calls were made by Ascent™ in the nine negative specimens, demonstrating a remarkably high specificity resulting in a negative percent agreement (NPA) of 100%. These results demonstrate the equivalence of Ascent™ to the gold standard of CMA/NGS in tissue for detecting chromosome arm-level aneuploidy, along with focal gene-level amplifications/deletions with high concordance in the same tissue.

### 3.2. Ascent™ Calls in CSF Demonstrate High PPA and NPA to Tissue-Based Tumor Profiling Results

**Figure 2:**
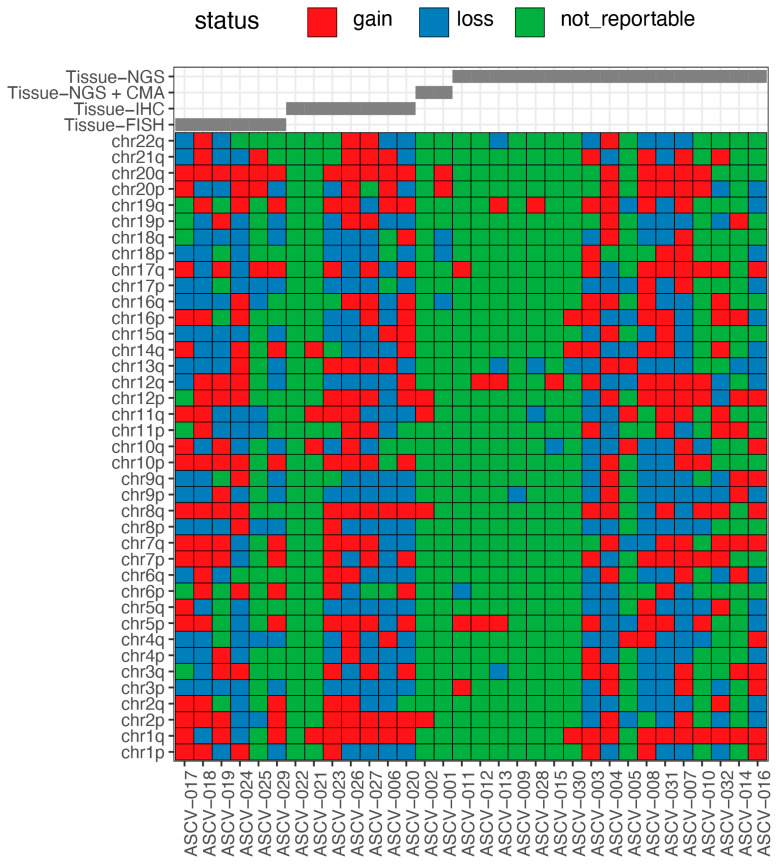
Aneuploidy plot of 32 samples in the validation cohort showing chromosomal arm-level loss/gains detected by Ascent™. Cohort had previous tumor profiling results using immunohistochemistry (IHC), chromosomal microarray (CMA), fluorescence in situ hybridization (FISH), or targeted next-generation sequencing (NGS). Legend: gain—increase in chromosome arm number; loss—deletion of chromosome arm; not reportable—no change in chromosome arm number. *X*-axis lists the specimen ID, and *Y*-axis shows the chromosome arm number evaluated.

Evaluation of Ascent™ data from 32 CSF specimens with accompanying tissue-based tumor profiling results from CMA/NGS/FISH/IHC (Table 3 and Figure 1C) resulted in 7 of 9 aneuploidy events in two CSF specimens being detected by Ascent™ for a PPA of 78% (Table 3 and Figure 1C). In 22 CSF specimens with only gene-level CNV events, Ascent™ detected 27 of 31 events, demonstrating 87% PPA relative to NGS and 100% PPA relative to IHC (detecting 4 of 4 events) and FISH (detecting 3 of 3 events) tumor profiling results (Table 3 and Figure 1C) for an overall PPA of 89% (detecting 34 of 38 events total) for gene-level CNVs. Additionally, in the eight specimens with no known aneuploidy or CNV events, Ascent™ showed 100% NPA, demonstrating high accuracy and specificity in detecting chromosome arm-level aneuploidy and gene-level CNVs in CSF specimens (Table 3 and Figure 1C). Further, a significantly high level of aneuploidy, indicative of chromosomal instability (>5 arm-level events, loss/gain), was observed in 73% (22 of the 30 specimens). Of these, two were evaluated with IHC, and seven were evaluated with NGS; all had previously undergone only targeted mutation testing in tumor tissue (Figure 2), pointing to the importance of evaluating chromosome aneuploidy and gene-level loss/gain using tests such as Ascent™ in the molecular testing algorithm for CNS cancers.

### 3.3. Ascent™ Informs Clinical Decision-Making as Demonstrated by the Detection of Chromosome Arm-Level Aneuploidy and Gene-Level CNVs Outlined in NCCN Guidelines

Based on the combined results from the equivalence (*n* = 48) and validation (*n* = 32) cohorts demonstrating a combined PPA of 94% for chromosomal arm-level loss/gain (detecting 32 of 34 events) and 93% for focal gene loss/gain (68 of 73) in 70 samples with 100% NPA, the impact of Ascent™ in the detection of chromosome arm-level loss/gain, as well as gene-level amplifications in primary and metastatic CNS cancers, was evaluated using 243 CSF specimens received for clinical testing that were positive for Ascent™ (Figure 1D). The primary CNS tumor cohort (*n* = 106) included 91 brain lesions and 13 primary CNS lymphoma and spinal cord tumor specimens (Figure 1D). The metastatic cohort (*n* = 137) included breast (*n* = 50), lung (*n* = 43), secondary lymphoma (*n* = 9), and a mix of other tumors (*n* = 35), such as kidney, prostate, etc. Chromosome arm-level aneuploidy was detected in 99% (136 of 137) of metastatic specimens and 86% (91 of 106) of primary specimens, of which gene-level CNVs were detected in 33% (45 of 136) metastatic specimens and 15% (14 of 91) were detected in primary specimens. Cumulatively, within the cohort of 243 specimens, chromosome arm-level aneuploidy was detected in 68% (164 of 243) of specimens, and gene-level CNVs were detected in 30% (72 of 243) of specimens (Figure 1D).

Chromosome arm-level aneuploidy and gene-level CNVs outlined in the adult NCCN guidelines [14], such as 1p/19q codeletion, co-occurrence of chromosome 7 gain and chromosome 10 loss, loss of 6q, monosomy 6, *CDKN2A/2B* deletion, *EGFR* amplification, *MYCN* amplification, and *ERBB2* amplification, were identified in multiple cases (Figure 1D). The results demonstrate the power of using Ascent™, which leverages the MethylsaferSeq [48] methodology for the evaluation of CNVs with high sensitivity, to inform the diagnosis and management of CNS tumors.

## 4. Discussion

Genomic characterization of cancer has become central to modern oncology diagnosis and therapeutic decision-making [49,50]. Detection of whole-chromosome and arm-level aneuploidy together with focal gene-level CNVs is now recognized as a core feature of cancer evolution, conferring growth advantage, treatment sensitivity/resistance, and worse survival in many advanced tumors [51,52]. In primary CNS tumors, specific events like 1p/19q co-deletion, *EGFR* amplification, *CDKN2A/2B* homozygous deletion, and +7/−10 are integral to the 2021 WHO classification, prognostication, and therapy decisions [5]. Also, brain metastasis occurs in nearly 20% of all patients with solid tumors, with the most common primary tissues of origin being breast, lung, and skin, in which the metastatic mass develop de novo aneuploidy patterns distinct from the parent tumor [53]. Karyotyping, CMA, NGS, and FISH are established reference methods for copy number and aneuploidy detection in tumor tissue specimens [25,54], while IHC is primarily a protein expression surrogate rather than a direct genomic assay, with recognized limitations in sensitivity and specificity [19]. CMA and NGS have been used in plasma to detect aneuploidy. However, CNS tumors pose unique diagnostic hurdles compared to extracranial cancers largely due to the BBB, which severely restricts the release of cell-free DNA into peripheral blood, resulting in low sensitivity for plasma-based liquid biopsies [55]. More importantly, brain surgery poses a significant risk to the patient, including hemorrhage, neurological injury, stroke, or even death [56], and the emergence of CNS-penetrant systemic therapies [57] underscores the need for CSF liquid biopsy to fast-track therapeutic decision-making in primary and metastatic CNS tumors.

To address the need for high-sensitivity tests that detect aneuploidy and focal gene CNVs in CSF, the equivalence of the Belay Ascent™ CSF-based liquid biopsy test for reliable detection of both chromosome arm-level aneuploidy and gene-level CNVs was evaluated, demonstrating performance comparable to established tissue-based methodologies. Ascent™ exhibited high concordance (PPA and NPA) with gold standard methods in tissue (CMA/NGS/IHC/FISH) (Figure 1), aligning with previous studies that established LP-WGS as a sensitive and cost-effective approach for genome-wide CNV detection in oncology [58,59]. The observed discrepancies in both the equivalence and validation cohorts in aneuploidy detection likely reflect both biological and technical factors. Tumor heterogeneity, temporal differences between tissue and CSF sampling, and the limited genomic scope of the CSF and tumor assays could contribute to discrepancies in aneuploidy detection [60,61,62]. Additionally, other biological and anatomical factors substantially affect assay sensitivity. Tumor size and overall disease burden are primary determinants of cfDNA levels in CSF. Anatomical location is also important; tumors in close proximity to CSF spaces, including leptomeningeal disease, are more likely to shed detectable DNA than those deeply situated parenchymal tumors or lesions with minimal CSF interface [63,64,65]. This is supported by the enrichment of high aneuploidy burden observed in metastatic cases with leptomeningeal involvement in the validation cohort. Furthermore, while approximately 90% of solid tumors exhibit aneuploidy indicative of chromosomal instability, the remaining ~10% of solid tumors that are diploid or near-diploid (euploid) possess distinct clinical and biological processes with varied prognostic implications [66,67,68,69].

Notably, Ascent™ identified increased aneuploidy (>5 events) in 73% of cases that had previously undergone only targeted tumor testing in tissue, highlighting a key advantage of genome-wide approaches compared to gene-specific marker-based assays. Ten of the eleven cases that had high aneuploidy were breast (four, of which three had LMD), lung (five, all had LMD), and sarcoma (one) metastatic cancers, with a single primary brain tumor. Aneuploidy burden has been associated with tumor aggressiveness, therapeutic sensitivity/resistance, and poor clinical outcomes across multiple cancer types, including primary CNS cancers such as glioma [70] and metastatic CNS cancers such as breast [7] and lung [8], particularly in cases with leptomeningeal disease, as observed in Ascent™ results in the validation cohort (Figure 2). While clinical diagnosis is provided in some cases (glioma, astrocytoma, etc., or tissue of origin such as lung or breast), additional stratification of the primary or metastatic cases was not feasible based on the limited clinical information obtained at the time of testing with regard to pathological diagnosis. The clinical utility of Belay tests was, however, recently demonstrated in a single-institution case series of 123 specimens, calling out the need to evaluate aneuploidy for comprehensive evaluation of metastatic CNS tumors, particularly those with a suspicion or confirmation of leptomeningeal disease [71]. Comprehensive assessment of chromosomal alterations may provide clinically relevant information that is not captured by limited gene panels or single-locus assays, such as those evaluated by FISH/IHC.

The equivalence and validation of the Ascent™ test in the detection of aneuploidy and gene-level CNVs are supported by its successful application in 243 real-world production cases. Given the cellular paucity in CSF, traditional methods like CMA, IHC, and FISH are often impractical or inconclusive for CNV and aneuploidy assessment in CNS tumors [72]. Ascent™ overcomes these limitations by enabling robust genome-wide analyses from minimal tumor DNA input, offering a minimally invasive alternative for patients who are not candidates for initial or repeat surgical resection or biopsy. These findings suggest that the CSF-based Ascent™ could either complement or, in certain clinical situations, be an alternative to tissue-based testing. This is especially relevant in cases of tumor recurrence, disease monitoring, or when surgical intervention poses significant risks [7,62,72], as well as in metastatic CNS cancers [7,8]. It is worth noting that LP-WGS usually requires a tumor fraction of 1–5% to reliably call aneuploidies in tissue [73]. However, its sensitivity and specificity, as demonstrated by Ascent™, are high in CSF, a specimen type known for its cellular paucity.

While LP-WGS of CSF offers a minimally invasive and sensitive approach for detecting chromosomal arm-level aneuploidy and copy number variations, a few practical limitations should be considered. Low-pass sequencing reduces per-sample sequencing costs compared to high coverage approaches; however, in terms of technical complexity, the workflow involves multiple steps, including CSF collection, nucleic acid extraction, library preparation, sequencing, and computational analysis, each of which may introduce variability and require standardized protocols to ensure reproducibility and reliable turnaround times [41,74]. Importantly, this approach is not designed to replace conventional diagnostic modalities, such as the pathological evaluation of tissue, where applicable. LP-WGS of CSF primarily provides genome-wide copy number information but lacks the resolution to define histologic tumor subtype or detect certain molecular alterations, such as point mutations, gene fusions, or epigenetic features, that are often required for definitive diagnosis and treatment stratification. Therefore, tissue-based histopathologic and molecular analyses remain necessary in most cases and should be considered complementary to CSF-based testing [41,74,75].

The unique proximity of CSF to the CNS allows for the detection of higher concentrations of circulating nucleic acids that are often undetectable in blood due to the blood–brain barrier, making CSF the specimen of choice for improved diagnostic accuracy [76,77,78,79,80,81,82]. To ensure higher sensitivity and specificity, Ascent™ was developed to be used in CSF independently and simultaneously with Summit™ 2.0 for the detection of chromosome aneuploidy for both primary and secondary CNS cancers, in line with the recommended guidelines. The test has been further validated to detect gene-level CNVs in CSF specimens and is best utilized when run simultaneously with a comprehensive genomic profiling test, such as Summit™ 2.0 [45], to better inform clinical decision-making.

## 5. Conclusions

In summary, this study demonstrates that LP-WGS-based Ascent™ is a reliable and minimally invasive test for detecting chromosome arm-level aneuploidy and gene–level CNVs in CNS malignancies. Additionally, the Ascent™ liquid biopsy test addresses a significant unmet need in neuro-oncology by overcoming the key limitations associated with plasma-based liquid biopsy and traditional cytogenetics methods. This makes it a valuable tool for diagnostic and therapeutic decision-making in primary and metastatic CNS malignancies.

## Figures and Tables

**Table 1:** Merits and demerits of methodologies for detecting chromosome aneuploidy and gene-level CNVs.

Methodology	Specimen Type	Biomarker/Target Evaluated	Merits	Demerits
Karyotyping	Tissue, cells	Chromosome aneuploidy	Visualization of structural changes	Restricted to chromosome aneuploidy
Immunohistochemistry (IHC)	Tissue	Targeted protein expression	Cost-effective, quick detection of key biomarkers	Limited specificity as gene expression does not directly indicate CNV
Fluorescence in situ hybridization (FISH)	Tissue	Rearrangements, targeted CNV evaluation	Reliable detection of key biomarkers	Targeted testing provides a limited molecular profile
Chromosomal microarray (CMA)	Tissue, blood	Chromosome aneuploidy	High-resolution, genome-wide CNV profiling	Low sensitivity in CSF due to high input DNA requirements
Next-generation sequencing (WGS)	Tissue, plasma	Chromosome aneuploidy	Genome-wide CNV profiling	Low sensitivity for gene-level CNV
Belay Ascent™ (low-pass whole-genome sequencing, LP-WGS)	CSF	Chromosome aneuploidy and gene-level CNVs	Low input DNA (<20 ng)	Longer turnaround times compared to IHC and FISH

CNV—copy number variants, CSF—cerebrospinal fluid, DNA—deoxyribonucleic acid.

**Table 2:** Equivalence of chromosome arm-level and gene-level variants detected by Ascent™ compared to CMA/NGS calls in tissue.

Study ID	Methodology	Known Results (Aneuploidy Reported)	Ascent (Aneuploidy Detected)	Known Results (CNVs Present)	Ascent (CNV Detected)
ASEV-001	CMA/NGS	None	None	None	None
ASEV-002	CMA/NGS	1p/19q Codeletion	1p/19q Codeletion	None	None
ASEV-003	CMA/NGS	None	None	EGFR amp, MDM4 amp, MYCN amp	EGFR (log2: 2.9), MDM4 (log2: 1.6), MYCN arm level (Log2: 0.13)
ASEV-004	CMA/NGS	None	None	None	None
ASEV-005	CMA/NGS	None	None	None	None
ASEV-006	CMA/NGS	1p/19q Codeletion	1p/19q Codeletion	None	None
ASEV-007	CMA/NGS	1p/19q Codeletion	1p/19q Codeletion	None	None
ASEV-008	CMA/NGS	1p/19q Codeletion	1p/19q Codeletion	CDKN2A/B (p16) (Loss)	chr9p21.3 (log: −0.7): CDKN2A, CDKN2B
ASEV-009	CMA/NGS	Gain of 7, loss of 10, 9pdel	Gain of 7, loss of 10, 9pdel	WT1 amp	Chr11p15.5-p11.1 (log2: 2.88): WT1 amp (p11.13)
ASEV-010	CMA/NGS	Gain of 7, loss of 10	Gain of 7, loss of 10	4q amp	4q amp (log2: 1.09)
ASEV-011	CMA/NGS	None	None	BRAF amp, MET amp, CDKN2A/B (p16) (Loss)	BRAF amp (log2: 0.4), MET amp (log2: 0.4), CKDN2A/B del (log2: −1.57)
ASEV-012	CMA/NGS	1p/19q Codeletion	1p/19q Codeletion	None	None
ASEV-013	CMA/NGS	1p/19q Codeletion	1p/19q Codeletion	None	None
ASEV-016	CMA/NGS	None	None	None	None
ASEV-017	CMA/NGS	None	None	CDKN2A/B (p16) (Loss)	chr9p21.3 (log: −0.9): CDKN2A, CDKN2B
ASEV-018	CMA/NGS	None	None	CDKN2A/B (p16) (Loss)	chr9p21.3 (log: −0.4): CDKN2A, CDKN2B
ASEV-019	CMA/NGS	None	None	CDKN2A/B (p16) (Loss)	chr9p21.3 (log: −0.611): CDKN2A, CDKN2B
ASEV-020	CMA/NGS	None	None	CDKN2A/B (p16) (Loss)	chr9p21.3 (log: −0.755): CDKN2A, CDKN2B
ASEV-021	CMA/NGS	None	None	None	None
ASEV-022	CMA/NGS	1p/19q Codeletion	1p/19q Codeletion	None	None
ASEV-023	CMA/NGS	None	None	CDKN2A/B (p16) (Loss)	chr9p21.3 (log: −0.643): CDKN2A, CDKN2B
ASEV-024	CMA/NGS	1p/19q Codeletion	1p/19q Codeletion	None	None
ASEV-025	CMA/NGS	None	None	CDKN2A/B (p16) (Loss)	chr9p21.3 (log: −0.513): CDKN2A, CDKN2B
ASEV-026	CMA/NGS	None	None	None	None
ASEV-027	CMA/NGS	1p/19q Codeletion	1p/19q Codeletion	None	None
ASEV-028	CMA/NGS	None	None	CDKN2A/B (p16) (Loss)	chr9p21.3 (log: −0.317): CDKN2A, CDKN2B
ASEV-029	CMA/NGS	None	None	None	None
ASEV-030	CMA/NGS	None	None	None	None
ASEV-031	CMA/NGS	None	None	CDKN2A/B (p16) (Loss)	chr9p21.3 (log: −2.19): CDKN2A, CDKN2B
ASEV-032	CMA/NGS	None	None	CDKN2A/B (p16) (Loss)	chr9p21.3 (log: −2.8): CDKN2A, CDKN2B
ASEV-033	CMA/NGS	1p/19q Codeletion	1p/19q Codeletion	None	None
ASEV-034	CMA/NGS	None	None	CDKN2A/B (p16) (Loss)	chr9p21.3 (log: −1.08): CDKN2A, CDKN2B
ASEV-035	CMA/NGS	None	None	None	None
ASEV-036	CMA/NGS	None	None	CDKN2A/B (p16) (Loss)	chr9p21.3 (log: −1.6): CDKN2A, CDKN2B
ASEV-037	CMA/NGS	1p/19q Codeletion	1p/19q Codeletion	None	None
ASEV-038	CMA/NGS	None	None	CDKN2A/B (p16) (Loss)	chr9p21.3 (log: −1.3): CDKN2A, CDKN2B
ASEV-039	CMA/NGS	1p/19q Codeletion	1p/19q Codeletion	CDKN2A/B (p16) (Loss)	CDKN2A, CDKN2B:Not detected (log2: −0.02)
ASEV-040	CMA/NGS	Gain of 7, loss of 10	Gain of 7, loss of 10	CDK4 amp, EGFR amp, KIT amp, MDM2 amp, PDGFRA amp	CDK4 amp (arm-level log2: −0.60 FC: 19.66), EGFR amp (FC: 7.59, Log2: 2.53), KIT amp (FC: 8.87, Log2: 1.02), MDM2 amp (arm-level log2: −0.60 FC: 24.61), PDGFRA amp (FC: 7.73, Log2: 1.02)
ASEV-041	CMA/NGS	None	None	None	None
ASEV-042	CMA/NGS	None	None	BRAF amp, MET amp, CKDN2A/B del	BRAF amp (arm-level log2: 0.48, FC: 2.32), MET amp (FC: 2.2, Log2: 0.44), CKDN2A/B del (log2: −0.825)
ASEV-043	CMA/NGS	1p/19q Codeletion	1p/19q Codeletion	None	None
ASEV-044	CMA/NGS	Gain of 7, loss of 10	Gain of 7, loss of 10	CDKN2A/B (p16) (Loss)	chr9p21.3 (log: −0.214): CDKN2A, CDKN2B
ASEV-045	CMA/NGS	None	None	MYCN amp	MYCN amp (arm-level log2: 0.2 FC: 1.32)
ASEV-046	CMA/NGS	1p/19q Codeletion	1p/19q Codeletion	None	None
ASEV-047	CMA/NGS	Gain of 7, loss of 10	Gain of 7, loss of 10	CDK4 amp, EGFR amp, MDM2 amp	CDK4 amp (FC: 38.69; log2: 0.07), EGFR amp (FC: 39.59; log2: 2.85), MDM2 amp (61.13; log2: 0.07)
ASEV-014	CMA	Complex genotype, very high chromosomal instability	1q−, 21q+, 5p+, 5q+, 7q+, 9p+, 9q+, 11p+, 11q+, 12p−, 12q−, 15q+, 16q−, 17p−, 17q−, 19p+, 19q+	None	None
ASEV-015	CMA	Complex genotype, very high chromosomal instability	1p+ 3p− 3q+ 4p− 4q− 5p+ 5q− 6p+ 6q+− 7p, 7q+ 8q+ 9p+ 9q+ 11p+ 11q+ 12p+ 12q+ 13q− 14q− 15q+ 17p− 18q + 19p+ 19q+ 1q+ 20p+ 20q+ 21q+ 22q−	None	None
ASEV-048	CMA	Complex genotype, very high chromosomal instability	2p+ 2q− 3p− 3q− 4p− 4q− 5p+ 5q+ 6p+ 6q+ 7p+ 7q+ 8p− 8q− 9p− 19q− 10p− 10q− 11p− 11q− 12p+ 12q+13q− 14q− 15q− 16p− 16q− 17p+ 17q+ 18p− 19p− 19q− 1p+ 1q+ 20p− 20q− 21q−	None	None

Chromosome microarray, CMA; next-generation sequencing, NGS; red font—variants not detected.

**Table 3:** Validation cohort (*n* = 32)—demonstrating correlation between Ascent™ calls in CSF with accompanying tumor profiling results.

Study ID	Primary/Metastatic	Tissue of Origin	Methodology	Aneuploidy/CNVs Reported in Tumor Profiling Results	Aneuploidy/CNVs Detected by Ascent
ASCV-002	Primary	Brain	Tissue-NGS + CMA	12+, 21+, 22q−, 2p+, 4+, 5p−, 8q+	2p+, 8q+, 11q+, 12p+
ASCV-001	Primary	Brain	Tissue-NGS + CMA	chr16q loss, chr20 gain,	chr16q loss, chr18p Loss chr18q Loss chr20p Gain chr20q Gain
ASCV-017	Metastatic	Breast	Tissue-FISH	*ERBB2* Amp	chr17q12 gain (*ERBB2*, Log2: 0.34)
ASCV-018	Metastatic	Breast	Tissue-FISH	*ERBB2* amp	chr17q12 loss (*ERBB2* Log2: −0.37)
ASCV-019	Metastatic	Lung	Tissue-FISH	*MET* Positive	chr7q31 (*MET* Log2: 0.40)
ASCV-024	Metastatic	Esophagus	Tissue-FISH	No CNVs	No CNVs
ASCV-025	Metastatic	Breast	Tissue-FISH	No CNVs	No CNVs
ASCV-029	Metastatic	Lung	Tissue-FISH	No CNVs	No CNVs
ASCV-022	Metastatic	Breast	Tissue-IHC	*ERBB2* amp	chr17q12 Gain (*ERBB2*)
ASCV-021	Metastatic	Breast	Tissue-IHC	*ERBB2* amp	chr17q12 Gain (*ERBB2*)
ASCV-023	Metastatic	Breast	Tissue-IHC	Her2 Positive	chr17q12 Gain (*ERBB2*)
ASCV-020	Metastatic	Breast	Tissue-IHC	*ERBB2* Amp	chr17q12 gain (*ERBB2*)
ASCV-026	Metastatic	Breast	Tissue-IHC	No CNVs	No CNVs
ASCV-027	Metastatic	Breast	Tissue-IHC	No CNVs	No CNVs
ASCV-006	Metastatic	Breast	Tissue-IHC	IHC-HER2 normal.	No CNVs
ASCV-011	Metastatic	Breast	Tissue-NGS	*FGFR1* amplification	chr8p11.23 gain (*FGFR1*)
ASCV-012	Metastatic	Lung	Tissue-NGS	*MDM2* amp	chr12q Gain (*MDM2* arm-level gain)
ASCV-009	Primary	Brain	Tissue-NGS	*EGFR* amplification, *CDKN2A/2B* deletion	chr7p11.2 Gain (*EGFR*), chr9p21.3 Loss (*CDKN2A/2B*)
ASCV-015	Metastatic	Lung/Breast	Tissue-NGS	*PTEN* loss	chr10q Loss (*PTEN*)
ASCV-028	Metastatic	Breast	Tissue-NGS	No CNVs	No CNVs
ASCV-030	Metastatic	Breast	Tissue-NGS	No CNVs	No CNVs
ASCV-013	Metastatic	Lung	Tissue-NGS	*MDM2* amp	chr12q (*MDM2* arm-level gain)
ASCV-003	Metastatic	Lung	Tissue-NGS	APC Loss; *CDNKA* Loss; *MTAP* Loss	chr17q12 gain (*ERBB2*), chr7p11.2 (*EGFR*), chr9p21.3 loss (*CDKN2A/B, MTAP*)
ASCV-004	Metastatic	Breast	Tissue-NGS	*AURKA* Gain, *GNAS* Gain	chr20q gain (*GNAS, AURKA*) chr7p11.2 Gain (*EGFR*)
ASCV-005	Metastatic	Lung	Tissue-NGS	*CCND1* amp, *MDM2* amp, *FGF19* amp, *FGF3* amp, *FGF4* amp,	chr11q gain (CCND1, FGF19, FGF3, FGF4 arm-level gain)
ASCV-008	Metastatic	Lung	Tissue-NGS	*CDK4* amp, *MDM2* amp	chr17q12 gain (*ERBB2*), chr7p11.2 gain (*EGFR*), chr12q gain (*CDK4, MDM2* arm-level gain)
ASCV-031	Metastatic	Lung	Tissue-NGS	*CDKN2A & CDKN2B* deletion	chr9p21.3 loss (*CDKN2A/B, MTAP*)
ASCV-007	Metastatic	Sarcoma	Tissue-NGS	*CDKN2A/2B* loss (NGS), SMARCA2 loss (IHC)	chr17q12 gain (*ERBB2*), chr9p21.3 loss (*CDKN2A/B*)
ASCV-010	Metastatic	Breast	Tissue-NGS	*ERBB2* amp, RAD21 amp	chr7p11.2 Gain (*EGFR*), chr17q12 Gain (*ERBB2*)
ASCV-032	Metastatic	Lung	Tissue-NGS	*MYC* copy number gain	Chr8q24.21 (*MYC* gain)
ASCV-014	Metastatic	Breast	Tissue-NGS	*PTEN* loss	chr10q23.31 Loss (*PTEN*)
ASCV-016	Primary	Brain	Tissue-NGS	*PTEN* Loss, *PDGFRA* Gain, *KIT* Gain,	chr4q12 gain (*PDGFR, KIT*) chr10q23.31 loss (*PTEN*)

Chromosome microarray, CMA; next generation sequencing, NGS; fluorescence in situ hybridization, FISH; immunohistochemistry, IHC; purple font—variants detected by Ascent known to be present in tumor; red font—variants known to be present in tumor, not detected by Ascent.

## Data Availability

All available data are included in this manuscript.
